# Molecular Imaging: A Useful Tool for the Development of Natural Killer Cell-Based Immunotherapies

**DOI:** 10.3389/fimmu.2017.01090

**Published:** 2017-09-12

**Authors:** Prakash Gangadaran, Byeong-Cheol Ahn

**Affiliations:** ^1^Department of Nuclear Medicine, Kyungpook National University School of Medicine and Hospital, Daegu, South Korea

**Keywords:** bioluminescence, cell trafficking, molecular imaging, natural killer cell, positron-emission tomography, single photon-emission computed tomography, therapy

## Abstract

Molecular imaging is a relatively new discipline that allows visualization, characterization, and measurement of the biological processes in living subjects, including humans, at a cellular and molecular level. The interaction between cancer cells and natural killer (NK) cells is complex and incompletely understood. Despite our limited knowledge, progress in the search for immune cell therapies against cancer could be significantly improved by dynamic and non-invasive visualization and tracking of immune cells and by visualization of the response of cancer cells to therapies in preclinical and clinical studies. Molecular imaging is an essential tool for these studies, and a multimodal molecular imaging approach can be applied to monitor immune cells *in vivo*, for instance, to visualize therapeutic effects. In this review, we discuss the usefulness of NK cells in cancer therapies and the preclinical and clinical usefulness of molecular imaging in NK cell-based therapies. Furthermore, we discuss different molecular imaging modalities for use with NK cell-based therapies, and their preclinical and clinical applications in animal and human subjects. Molecular imaging has contributed to the development of NK cell-based therapies against cancers in animal models and to the refinement of current cell-based cancer immunotherapies. Developing sensitive and reproducible non-invasive molecular imaging technologies for *in vivo* NK cell monitoring and for real-time assessment of therapeutic effects will accelerate the development of NK cell therapies.

## Introduction

Over the past few decades, imaging has changed the way medicine is practiced. Recent advances in molecular imaging allow the visualization of both cellular and subcellular biological and pathophysiological processes within living subjects (1). It is important to assess the efficiency of various cell therapies in certain tumors, to identify the most effective application in a given clinical context. Molecular imaging has emerged as a new technology for development of both research and clinical cell-based therapies. Investment in molecular imaging technologies is expected to enhance the efficacy of cell-based therapies (2, 3). Molecular imaging generally exploits specific molecular probes, as well as intrinsic tissue characteristics, as the source of image contrast, and provides insight into integrative biology, disease characteristics, early disease detection, and therapeutic efficacy (4). Furthermore, it offers the possibility of repetitive, uniform, non-invasive, and comparatively automated studies of living subjects, using identical or alternating biological imaging assays, at different time points, thereby harnessing the statistical power of longitudinal studies and reducing the cost and number of animals required (2, 4). The advantage of molecular imaging over conventional anatomical imaging is that it can be performed in an intact organism with sufficient spatial and temporal resolution to study biological processes *in vivo*. Prior to clinical application, it is useful to assess the temporal and spatial biodistribution of imaging probes or drugs and their diagnostic and therapeutic efficiencies. Molecular imaging can provide this information in a preclinical animal model setting and even in certain clinical settings.

Immunotherapy is an innovative and promising approach to the treatment of cancer and is based on the idea of destroying malignancies by means of immune cells or stimulating the immune system to fight cancer (5, 6). The concept that the immune system can recognize and control tumor growth can be traced back to 1893, when William Coley used live bacteria as an immune stimulant to treat cancer (7). In recent years, scientific advances have expanded our understanding of the immune system and its response to malignant cells. The clinical goal of tumor immunotherapy is to provide either passive or active immunity against malignancies by harnessing the immune system to target tumors (8, 9). The immune system’s natural capacity to detect and destroy abnormal (cancer) cells may prevent the development of many cancers (10). In the past few decades, immunotherapy has become an important part of treatment for some types of cancers.

In the past decades, cell-based immunotherapy has represented an expanding segment of cancer treatment. There are a few immune cell types that play important roles in cancer therapies, including dendritic cells (11), T cells (12), B cells (13), and natural killer (NK) cells (14). NK cells were first identified in 1975 as a unique lymphocyte subset with cells that are larger in size than T and B lymphocytes and that contain distinctive cytoplasmic granules (14, 15). These cells are able to recognize and kill tumor cells without the need for prior treatment or antigen exposure. Since their discovery, NK cell-based immunotherapies have held great promise for cancer treatment. However, thus far, only limited clinical success has been achieved by using NK cell-based therapies in cancer patients (14, 16). Progress in understanding NK cell biology and function is therefore needed to enable the development of novel approaches to effectively manipulate NK cells for cancer treatments. Molecular imaging research is contributing to our understanding of NK cell-based therapies and is helping to extend more effective care to patients. The molecular imaging techniques used to image NK cells include fluorescent imaging, microscopic imaging, intravital imaging, bioluminescent imaging, radiotracer/nuclear imaging, magnetic resonance (MR) imaging, and others. In this article, the contribution of molecular imaging to the development of NK cell-based therapies, and comprehensive details of diverse molecular imaging modalities, will be discussed.

## NK Cells and NK Cell-Based Cancer Immunotherapy

Natural killer cells are derived from hematopoietic stem cells in the bone marrow; these lymphocytes are a major component of the innate immune system (16, 17). NK cells represent 5–20% of peripheral blood mononuclear cells and these cells can be subdivided into different populations, based on the relative expression of the surface markers CD16 and CD56. The two major subsets are CD56^bright^CD16^dim/−^ and CD56^dim^CD16^+^ (18, 19). They can directly induce apoptosis of infected cells *via* the perforin-granzyme pathway or by expressing death-receptor ligands, such as Fas ligand (20–22). The gain of NK cell cytotoxicity during evolution has been associated with the development of highly refined and robust mechanisms to control cytolysis to prevent tissue damage. NK cells do not rearrange their immune receptor genes or express T-cell antigen receptors (23). NK cells are activated by cytokines, such as interleukin (IL)-12, IL-15, IL-18, IL-2, and CCL5, which play pivotal roles in the maturation, activation, and survival of NK cells (24–26). IL-2 is one of the ideal cytokines required for NK cells to survive and proliferate (27). NK cell triggering is the result of a complicated balance between activatory and inhibitory signals; these triggers require deficiency of MHC-I expression on target cells (28, 29) and the expression of inducible ligands to activate NK cell receptors (30).

Natural killer cell line NK-92 was developed, in 1992, from isolated peripheral blood lymphocytes of a patient with large granular lymphoma (31). NK-92 cells showed very high cytotoxicity against diverse malignancies, both *in vitro* and *in vivo* (32). NK-92 cells show greater cytotoxicity than do other NK cell lines; it is the only NK cell line that is consistently and highly cytotoxic to cancer cell targets (33). NK-92 is currently the only NK cell line that has entered clinical trials and that can serve as a platform for studying NK cell-based tumor immunotherapy to date (14). This cell line proliferates and expands easily, with a doubling time of 4 days, and thus, the cells can be administered to patients repeatedly (34).

The high and selective cytotoxicity of NK cells to cancer cells offers a new therapeutic approach to avoid harming healthy cells, in the absence of preimmunization or stimulation (14, 32). NK cells play a critical role, both directly and indirectly, in the initial line of defense against tumors. NK cell activity is controlled by signaling *via* activatory and inhibitory receptors (35–37), and the clinical benefit of autologous NK cell therapy has been marginal, because of the limited activity of NK cells. Certain cytokines are able to activate NK cells, and systemic administration of these cytokines can induce apoptosis of tumor cells. However, severe side effects, including vascular leak syndrome, can result (14). Activated NK cells can be acquired by adoptive transfer, rather than systemic administration, of IL-2 (14), and, when combined with IFN-α, this approach has been shown effective (38). Allogeneic NK cells can be adoptively transferred to patients after *ex vivo* expansion and activation of unstimulated donor NK cells. This method showed greater tumor killing activity and was safe, with minimal toxicity. Therapies with allogeneic NK cells were attempted in treating various cancers, including melanoma, renal cell carcinoma, and lung cancer. Rejection of NK cells by a patient’s immune system is one of the causes for therapy failure (39–42).

Natural killer cells can be expanded *in vitro* whenever necessary, and expanded cells are safe to administer as monotherapy in patients with advanced digestive cancer (37). Furthermore, NK cell cytotoxicity is known to be excellent against melanoma and renal carcinoma cells (14). NK-92 cells have shown anticancer effects in tumors and have been demonstrated to be safe. Importantly, their antitumor activities can be enhanced, and large-scale production is possible making them amenable for use in clinical trials (14, 43). Overexpression of activating and inhibitory receptors might be effective in modulating and enhancing NK cell–tumor interactions. This gene modification approach resulted in a stronger intracellular cytotoxic signal and increased tumor cell killing by NK cells (32, 44, 45). Despite their successes, conventional histopathological and cytological methods have significant limitations when used in biological experiments. They usually require chemical fixation of excised tissues and the observation of biological samples under non-physiological conditions, which generally prevent resolution of the dynamics of the cellular processes. Most importantly, it is very difficult to generate quantitative data using conventional methods. Non-invasive *in vivo* imaging methods can show specific cellular and molecular processes. Molecular imaging allows monitoring of time-dependent experimental, developmental, environmental, and therapeutic effects of NK cell-based treatments in the same animal or patient.

## Overview on Molecular Imaging

Molecular imaging allows the non-invasive assessment of pathophysiological processes, which can inform proper decision-making in preclinical and clinical scenarios, which can help researchers accelerate the development of immune cell therapies, enhancing therapeutic efficacy and reducing adverse effects. Molecular imaging technologies have improved with the development of new reporter contrast agents, imaging agents, ligands, and probes. Molecular imaging techniques, such as fluorescence imaging, bioluminescent imaging, computed tomography, MR imaging, ultrasound, single photon-emission computed tomography (SPECT), and positron-emission tomography (PET) can be used effectively to track stem cells and immune cells for cancer treatments (46–51). Among the different molecular imaging techniques, optical imaging based on fluorescence and bioluminescence has shown the highest sensitivity in small animal studies. In addition to the benefit of an exceptionally high signal-to-noise ratio, optical molecular imaging provides multiplex imaging potential though the use of various probes with different spectral characteristics, and it requires relatively low-cost instrumentation (4). The advantage of optical bioluminescent imaging is that it allows detection of very low levels of signals from animals, in the absence of background signals.

Positron-emission tomography is a sensitive molecular imaging modality that can non-invasively assess cell retention, survival, and function after transplantation (52). Positron-labeled molecular probes, including ligands or substrates, bind to their specific target proteins or are trapped in cells of interest. The concentration of the positron-emitting probe is then measured with a PET scanner (53). SPECT imaging probes are labeled with gamma-emitting radionuclides [e.g., technetium-99m (^99m^Tc), indium-111 (^111^In), iodine-123 (^123^I), and iodine-131 (^131^I)], whereas PET tracers are labeled with positron-emitting radionuclides [e.g., oxygen-15 [^15^O], nitrogen-13 (^13^N), carbon-11 (^11^C), and fluorine-18 (^18^F)] (54). SPECT has an advantage over PET technology due to its multiplexing capability, the use of radionuclides that emit different gamma energies, the lack of the need for a cyclotron, and the low instrumentation cost. SPECT imaging has been widely used to monitor radionuclide-labeled cells in animals and human patients (55, 56). Magnetic resonance imaging (MRI) provides high-resolution anatomical information with excellent tissue contrast; however, MRI has low sensitivity for molecular detection as compared to other molecular imaging modalities, which is the major limitation of this technology (57). Optical imaging is suitable for tracking NK cells in small animals (58). Microscopy can give detailed information about molecular and cellular interaction of NK cells and is suitable for *in vitro* NK cells experiments; however, microscopy is unsuitable for non-invasive *in vivo* visualization and for tracking of cells in live animals and humans (48, 59). There are a few imaging modalities that can be used in humans to visualize NK cells: gamma camera imaging, SPECT, PET, and MRI. SPECT, PET, and MRI are capable of visualizing *in vivo* cell migration anywhere in the human body, as three-dimensional (3-D) imaging data. Each imaging modality has its own advantages and disadvantages; hence, the choice of imaging modality or combination of techniques must be determined based on the specific biological question.

## Tracking NK Cells by Molecular Imaging

**Figure 1:**
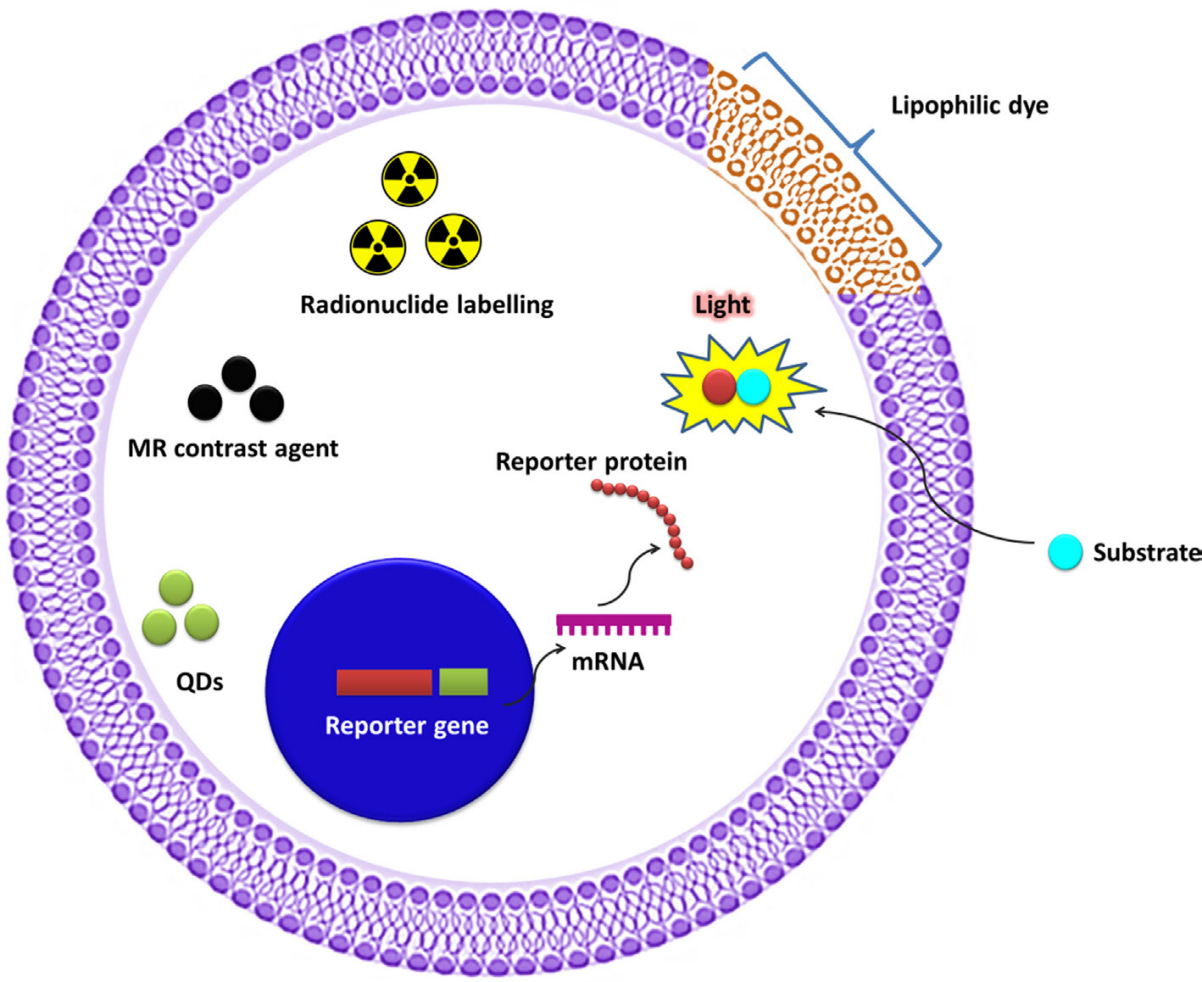
Strategy for labeling natural killer (NK) cells for molecular imaging.

**Figure 2:**
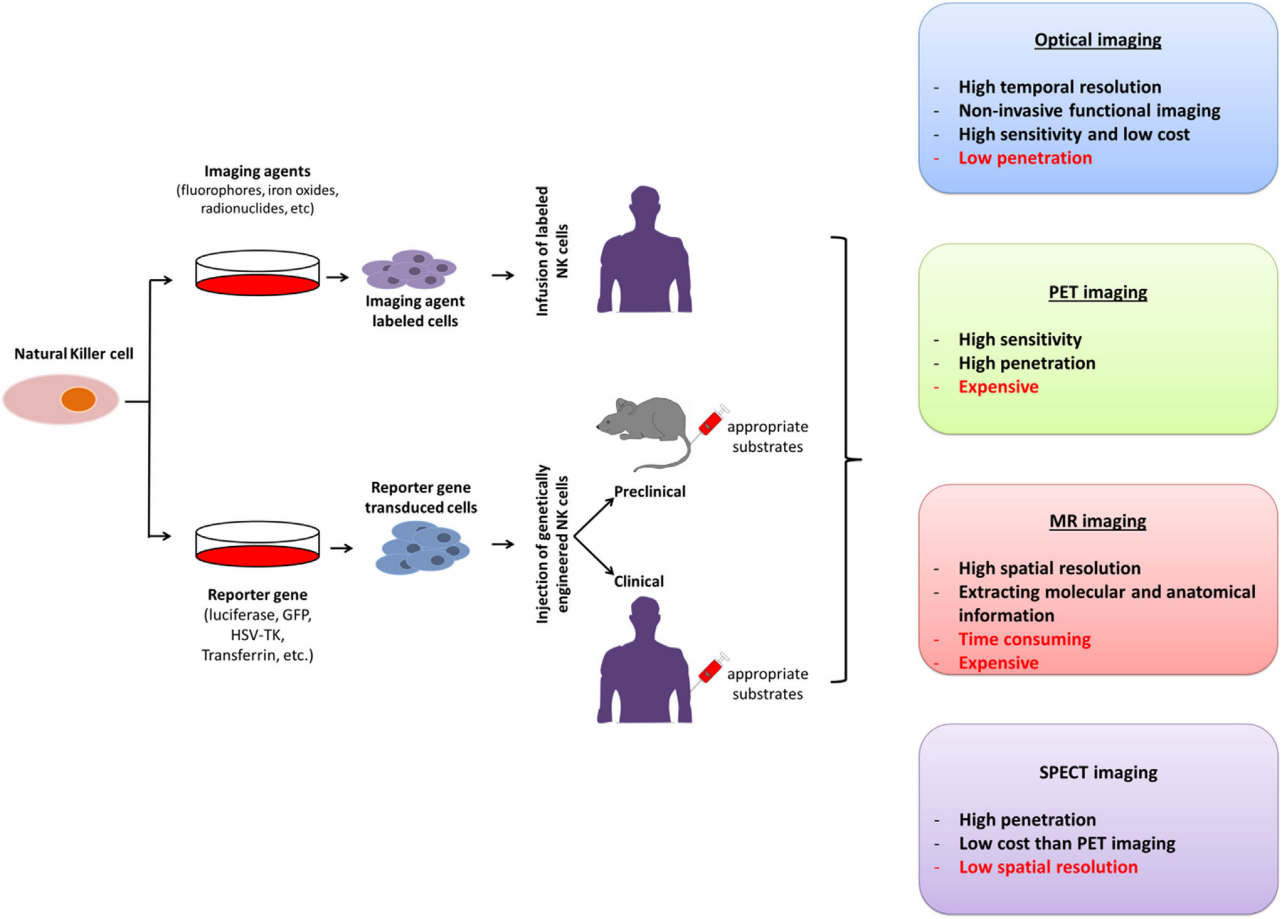
Molecular imaging strategies for non-invasive *in vivo* tracking of natural killer (NK) cells; their advantages and disadvantages. PET, positron-emission tomography; SPECT, single photon-emission computed tomography; MR, magnetic resonance; GFP, green fluorescence protein; HSV-TK, Herpes simplex virus-thymidine kinase.

The unique features of molecular imaging allow us to expand our knowledge of NK cell-based immunotherapies against cancers and provide bridges to clinical applications of these therapies. Therefore, molecular imaging is exceedingly useful in the development of NK cell-based immunotherapies. Molecular imaging is already well integrated into preclinical and clinical studies and has been used for therapy optimization. This application will eventually reduce the cost and time required for the development of NK cell-based immunotherapies for cancer. Over the past decade, advances in controlling immune cells have prompted the development of new immunotherapies. The development of clinically applicable methods for producing large numbers of fully functional NK cells is crucial for maximizing the potential of this therapeutic approach. Many studies have investigated the cytotoxic effects of NK cell immunotherapies in various cancers (10, 14, 31, 45, 60–62). Non-invasive imaging modalities can play key roles in studies by allowing visualization and quantification of real-time *in vivo* kinetics of NK cells after their administration and the biological status of the targeted diseases. Molecular imaging has been applied to many preclinical and clinical investigations of cell-based therapies. NK cell-labeling strategies and non-invasive NK cell tracking by means of molecular imaging are illustrated in Figures 1 and 2.

Natural killer cell tracking with optical imaging was successfully performed by transfecting cells with fluorescent or luciferase genes (63, 64). Expression of a receptor to a specific tumor antigen on NK cells might enhance homing of NK cells to tumors harboring the specific antigen. Generally fluorescent imaging approaches are inexpensive, fast, and are not associated with radiation exposure, and are amenable to panel studies (4, 65). Zhu et al. recently demonstrated the ability of NK92MI cells, expressing luciferase, to target anaplastic thyroid cells (CAL62) metastasized to the lung and subcutaneous tumors in a mouse model. This imaging approach further revealed that intravenously injected NK92MI cells localized mostly to the lung in the lung metastasis model and that signals were diminished after 2 days. The subcutaneous tumor-bearing mouse model showed that NK cells initially stayed in lung, but after 3 h, the cells reached the tumor implanted in the lower flanks of the mice. Signals were visible even after 2 days (66).

Tavri et al. detected increased fluorescent signaling in tumors at 24 h after injecting NK cells labeled with dioctadecyl tetramethylindodicarbocyanine perchlorate (DiD) fluorescent dye (64). They showed effective NK cell labeling and tracking by simple incubation with this commercially available lipophilic dye for a few minutes. This techniques is less complicated and less expensive than the previously applied labeling techniques that involved radionuclides (56, 61, 67), MR agents (68, 69), or reporter genes (63). However, in this study fluorescent imaging faced a challenge in terms of translational applications, as the technique demonstrated limited penetration depth (64). Lim et al. investigated human NK cells (NK92MI) labeled with anti-human CD56 antibody-coated quantum dots (QD705) and injected these directly into tumors to test their tumor cytotoxicity. The labeled and unlabeled NK cells showed similar cytotoxicity, suggesting that NK cells can be labeled with near infrared (NIR) dye without compromising their therapeutic effects (70). The advantages of QDs for biological applications include their high quantum yield, color availability, and good photostability. However, QDs are not readily internalized by NK cells, and thus there is a need to enhance the labeling efficiency to facilitate use of QDs in NK cell tracking.

Visualizing the microarchitecture of immune cells and the immunological synapses has been challenging, due to the limited resolution of microscopy technology. Microscopy technology has been developing steadily and has recently become available for exploration of the microarchitecture of immune cells. Treanor et al. have applied fluorescence lifetime imaging to reveal that killer Ig-like receptor (KIR) phosphorylation in NK cells (expressing KIR-GFP) was involved in contacting target cells in NK cell immune synapses; this further elucidated how NK cells respond appropriately when simultaneously surveying vulnerable and resistant target cells (71). Benninger et al. revealed the structure of NK cell immune synapses using the fluorescence imaging of two-photon linear dichroism. This imaging approach revealed that NK cell plasma membrane is extensively ruffled at the periphery, however not in the middle of the mature cytolytic NK cell immune synapses. Furthermore, Time-lapse imaging showed that the initial plasma membrane ruffles formation at the first point of interaction between NK cells and target cells, then the continues imaging showed that the plasma membrane ruffles formation spread radially across the intercellular contact when the size of the immune synapses augmented, finally disappeared from the center of the mature synapse (72).

Oddos et al. used optical tweezers along with a confocal microscope to image the immune synapses in live cells. Here, they used a combination of optical tweezers and confocal microscopy to achieve generally applicable, high-speed, high-resolution imaging, which is essential for the visualization of intercellular contacts (between NK cells expressing KIR-GFP and target cells labeled with a membrane dye); this dynamic imaging approach applied to image immune synapses in live-cell conjugates, which revealed clusters of effector cell (T cell) receptors at the antigen-presenting cells and long, receptor-rich filopodia structures within inhibitory NK cell immune synapses (73). Konishi et al. developed a novel *in vivo* molecular imaging tool to visualize the granzyme B activity using fluorescent molecular tomography in conjunction with coregistered computed tomography imaging (FMT-CT) and demonstrated that Granzyme B is the major effector of n CD8+ T cell-mediated myocarditis. These imaging approach of Granzyme B activity can permit non-invasive imaging of immune-mediated myocarditis *in vivo* and reveal therapeutic effect of pharmacologic interventions (74).

Chauveau et al. experimented with primary NK cells labeled with DiD or dioctadecyloxacarbocyanine perchlorate dye to explore the long-distance interaction between NK cells and target cells by applying quantitative live-cell fluorescence imaging. This imaging method demonstrated that nanotubes allow human NK cells to interact functionally with target cells over long distances; furthermore, the number of nanotubes developed was dependent on the number of receptor/ligand interactions, involving the activating receptor NKG2D, and MHC class I chain-related protein A (75). Brown et al. applied super-resolution microscopy to study the NK cell immune synapses and remodeling of cortical actin by using NK cells expressing GFP, mCherry, and LysoTracker Red dye (which allows labeling and tracking acidic organelles in live cells). The activation state of NK cells was visualized by three-dimensional-structured illumination microscopy, with a super-resolution of 100 nm, which revealed increased cortical actin mesh in the immune synapse. Furthermore, two-color super-resolution imaging revealed that lytic granules docked precisely in particular domains. Super-resolution microscopy has advanced the understanding of immune cell biology, and particularly NK cell biology, by exploring the structure of F-actin in NK cell immune synapses (76). A another study from Brown et al. also used super-resolution microscopy to study the F-actin, lytic granules, and IFN-γ in stimulated primary human NK cells through different activating receptors. This microscopic imaging approach revealed that lytic granules and IFN-γ accumulated in domains where the periodicity of the cortical actin mesh at the synapse opened up to be penetrable (77).

Tuli et al. used fixed-cell confocal microscopy to explore the formation of immune synapses between NK and target cells and the polarization of NK cells. They used GFP- and YFP-transduced NK cells and target cells membrane-labeled with PKH26 (red) or PKH67 (green) dyes and demonstrated that ADP-ribosylation factor-like 8b (Arl8b) regulates lytic granule polarization and NK-mediated cytotoxicity (78). Viswanathan et al. applied confocal microscopy imaging to demonstrate NK cell polarization by labeling NK and target cells with Lysotracker Red DND-99 and Cell Mask Deep Red, respectively. Furthermore, they also visualized synaptic granule externalization (79). Hsu et al. used live-cell and fixed-cell confocal microscopy, with NK cells and YTS cells (hybridoma) labeled with LysoTracker Red DND-99 dye, to study the promotion of cytotoxicity and prevention of bystander killing. This microscopy approach revealed that NK cell cytotoxicity granules avoid damaging or killing neighboring healthy cells (80). Rak et al. applied several microscopies (total internal reflection fluorescence microscopy, confocal microscopy, stimulated-emission depletion microscopy and platinum replica electron microscopy) to evaluate the relationship between the actin network and degranulation. These microscopic approaches revealed that actin filaments are existing all over the synapse and that NK cells overcome the actin obstacle not by comprehensively clearing but by allowing minimally sufficient conduits in the actin network. Furthermore, their result provides evidence of F-actin as an enhancer of secretion rather than an obstacle; it would have been not know without using very high-resolution imaging technique used in this study (81).

To address how NKG2D engagement affects intratumoral NK cell dynamics, Deguine et al. performed intravital two-photon imaging, using NK cells expressing GFP in a Rae-1b-expressing solid tumor mouse model. The intravital imaging showed higher intratumoral NK cell density and dissemination by Rae-1b expression. Furthermore, they imaged interactions of NK cells with target cells (MHC-deficient and peptide-pulsed solenocytes, respectively) in lymph nodes (50). The cytolytic activity of NK cells is assessed either through a degranulation assay (LAMP1/CD107a) (82) and/or through a cytotoxicity assay. One alternative method based on a non-toxic fluorescent dye using Calcein AM (acetoxymethyl) was developed in 1994 (83). Somanchi et al. used fluorescence imaging to assess NK cell cytotoxicity against neuroblastoma and leukemia. Fluorescence images used to overcome the limitation of the calcein release variation and detect the unreleased calcein within apoptotic bodies of lysed tumor targets or incomplete release (84).

Vanherberghen et al. used a microchip-based, time-lapse imaging approach and recorded the entire contact history of each NK cell. Using this imaging approach they quantify the cytotoxic response of individual NK cells; further they also quantified that 50% NK cells did not kill any target cells and small population of NK cells are responsible for most of the target cell deaths. They classified the portion of NK cell population as “serial killers” (85). Chatzopoulou et al. used a chip-based imaging platform to analysis the dynamics of NK cell cytolysis. They particularly used a fluorescent indicator of cell death and time-lapse microscopy and measured specific target cell lysis by activated human NK cells. Compared to the classical calcein assay, the chip-based approach has advantages of high sensitivity and long term assessment (86).

Beuneu et al. demonstrated that NK cell activation in lymph nodes can be imaged using NK cells and DCs expressing different fluorescent proteins with confocal and two-photon imaging in a mouse model. In addition, they showed that T cells and NK cells use different modes of interactions with stimulatory DCs (87). Bajénoff et al. used dynamic intravital imaging to investigate NK cell localization and behavior in lymph nodes in a *Leishmania major*-infected mouse model with fluorescent-tagged antibodies. Intravital microscopy imaging demonstrated location of NK cells in the outer regions of lymph nodes of the infected mice; the organisms slowed slow motility and interacted with DCs for a prolonged time (88).

Meier et al. labeled genetically engineered NK cells (expressing a receptor for prostate cancer) with iron oxide (ferumoxides) and visualized the *in vivo* kinetics of the NK cells by MRI. The group demonstrated increased prostate cancer targeting of the genetically engineered NK cells (89). They observed a drastic decline in tumor signals displayed by NK-92 cells labeled with ferumoxides and lipofectin; this was because the labeling methods used in this study was not suitable for long-term monitoring, and thus the time points they studied was short (24 h). Similarly, in another study, NK cells artificially expressing a receptor for breast cancer cells was established, and their biodistribution was assessed by MRI after labeling cells with ferucarbotran and ferumoxides by lipofection and electroporation, but not by simple incubation. Since they used clinically applicable contrast agents, this approach was suitable for clinical applications. The genetically engineered NK cells progressively accumulated in breast tumors by 12 and 24 h (90). Thus, the ability of therapeutic NK cells to target certain diseases can be assessed by MRI, and an MRI platform can be used for functional evaluation of genetic engineering of NK cells. However, the sensitivity of MRI is markedly lower than that of PET, SPECT, or optical technologies; therefore, advancements in terms of enhancing the sensitivity of MRI are urgently needed to improve application of this technology in preclinical and clinical studies.

Melder et al. showed by PET imaging that tumors preferentially take up activated NK cells (91), when NK cells were isolated from mice and labeled with ^11^C-methyl iodide. In this study, 4–30% of activated NK cells were retained in tumors, whereas non-activated NK cells were not retained in tumors. The labeled NK cells could be visualized within tumors as small as 2 mm in length by PET imaging. PET imaging demonstrated that activated NK cells can be retained preferentially in tumors without requiring the sacrifice of the experimental animals (91). PET provides the advantages of high sensitivity, three-dimensional resolution and absolute quantification in animal or human subjects, in a non-invasive manner. Galli et al. revealed NK cell infiltration into tumorous lesions using gamma camera imaging with ^99m^Tc-anti-CD56 monoclonal antibodies (mAbs) in mice with anaplastic thyroid cancer. Because of the long half-life of ^99m^Tc (6 h) as compared to that of ^11^C (20 min), NK cells in tumors can be assessed with ^99m^Tc-anti-CD56 mAb over a period of 24 h, demonstrating the potential for SPECT tracers as a gamma probe to image NK cells *in vivo* (92). This approach overcomes certain limitations, such as that ^111^In-oxine may have detrimental effects on NK cells and is rapidly released from labeled NK cells (61, 93) and have been proven to be suitable for visualizing NK cells in an *in vivo* animal model. However, this may not reflect the situation in humans.

Matera et al. showed that ^111^In-oxine labeled NK cells adoptively transferred to the liver *via* the intra-arterial route have preferential access to and accumulate substantially in hepatic metastases of colon carcinomas in a clinical scenario, whereas intravenously injected NK cells first accumulated in the lung and then in the spleen and liver. Migration of these cells to various organs was also evaluated by SPECT (93). Meller et al. showed the biodistribution and tracked the migration of NK cells in patients with renal cell carcinomas with SPECT imaging using ^111^In-oxine labeled NK cells. Initial imaging revealed that the cells accumulated in the lungs and were later redistributed to the liver, spleen, and bone marrow. The tracer accumulated in two of four large metastases, as shown by SPECT imaging. This study could not definitively clarify the intraorgan fate of the injected NK cells. Nevertheless, the injected NK cells could be detected up to 72 h by labeling with ^111^In-oxine, due to the long half-life of ^111^In (67 h), and accumulation of the labeled NK cells in tumors is one of major factors in the anti-tumor effect of the cell-based therapy *in vivo*. Uptake of ^111^In-oxine-labeled NK cells was only detectable in large tumor sites with high glucose metabolism, but not all large tumor lesions with high glucose metabolism demonstrated this uptake of NK cells (61). Meier et al. showed that NK-92 cells and NK-92-scFv(FRP5)-zeta cells were labeled with [^18^F]FDG by simple incubation under different conditions. After injection of 5 × 10^6^ [^18^F]FDG-labeled NK-92-scFv(FRP5)-zeta cells into tumor-bearing mice, digital autoradiography showed an increased uptake of radioactivity in HER2/neu-positive tumors at 60 min postinjection; NK-92 cells labeled with [^18^F]FDG did not result in increased uptake of radioactivity in the tumors on digital autoradiography (67). This technique would not be suitable for the long-term monitoring of injected NK cells that accumulate in the tumor tissue, due to short half-life of ^18^F. However, the advantage of [^18^F]FDG is that it is an FDA-approved radiopharmaceutical; translation into the clinical environment is relatively straightforward. Examples of *in vivo* monitoring of NK cell-based therapies for cancers with molecular imaging are summarized in Table 1.

**Table 1:** Molecular imaging strategies for *in vivo* natural killer (NK) cell tracking.

Imaging modality	Cell type	Cell origin	Labeling method	Subject	Route of injection	Duration of tracking	Purpose	Clinical translation	Reference
FLI	NK-92	Human	NIR dye	Rat	Intravenous	24 h	Tracking	Limited	(64)
FLI	NK92MI	Human	NIR dye	Mouse	Intratumor	Immediate	Therapy	Limited	(70)
BLI	Primary cell	Mouse	Fluc	Mouse	Intravenous	0–12 days	Tracking	Limited	(63)
BLI	NK-92MI	Human	Fluc	Mouse	Intravenous	0–72 h	Tracking and therapy	Limited	(66)
MRI	NK-92	Human	Ferumoxides	Rat	Intravenous	1–24 h	Tracking and therapy	Yes	(89)
MRI	NK-92MI	Human	SPIO	Rat	Transcatheter	Immediate	delivery	Yes	(68)
MRI	NK-92	Human	Ferumoxides	Mouse	Intravenous	12 and 24 h	Tracking and therapy	Yes	(90)
PET	Primary cell	Mouse	^11^C	Mouse	Intravenous	0.5–1 h	Tracking	Yes	(91)
Gamma camera	Primary cell	Human	^99m^Tc	Mouse	Intravenous	1–24 h	Infiltration into tumor	Yes	(92)
SPECT	Primary cell	Human	^111^In	Human	Intravenous	0.5–144 h	Tracking and therapy	Yes	(61)
SPECT	Primary cell	Human	^111^In	Human	Intravenous	6 days	Biodistribution	Yes	(61)
SPECT	Primary cell	Human	^111^In	Human	Intra-articular and venous	6–96 h	Tracking	Yes	(93)
Autoradiography	NK-92	Human	[^18^F]FDG	Mouse	Intravenous	0.5 h	Tracking to tumor	Yes	(67)

*NK-92 is a continuously growing cell line that has features and characteristics of NK. The cells came from a patient who had an NK cell lymphoma. NK92MI, an interleukin-2 (IL-2)-independent NK cell line derived from the NK-92 cell line*.

*FLI, fluorescence imaging; PET, positron-emission tomography; SPECT, single photon-emission computerized tomography; BLI, bioluminescence imaging; MRI, magnetic resonance imaging; NIR, near infrared; QD, quantum dot; SFB, fluorobenzoate; SPIO, superparamagnetic iron oxide; Fluc, firefly luciferase*.

## NK Cell Cytotoxicity Monitoring by Molecular Imaging

Natural killer cells are programmed to kill target cells. The molecular mechanism underlying NK cell killing is very complex, with NK cells displaying cytotoxicity through two major mechanisms. First, NK cells induce cell death through perforin/multiple granzyme-dependent necrosis; second, NK cells induce apoptosis through at least one of three death ligands (TNF-α, FasL, and TRAIL), each of which interacts with specific receptors on target cell surfaces (94, 95). We have previously demonstrated the therapeutic effects of NK cells *in vitro* on doxorubicin-sensitive and -resistant breast cancer cells, with *in vitro* molecular imaging using bioluminescent reporter genes (94). One of the limitations of this study was the lack of *in vivo* results regarding NK cell cytotoxicity in breast cancer tumors. In another study, we showed the *in vivo* therapeutic efficiency of NK cells in breast cancer cells by means of *in vivo* molecular imaging using bioluminescent reporter genes (96).

Apoptosis is a major mechanism through which NK cells induce programmed cell death of target cells. This involves several molecular events, such as the release of cytochrome c, formation of an apoptosome, and activation of effector caspase-3, -6, and -7 (22, 97). Lee et al. non-invasively visualized early cellular apoptotic events induced by NK cell therapy both *in vitro* and *in vivo* using a caspase-3 biosensor with *Renilla* luciferase. Edinger et al. non-invasively assessed lymphoma growth and the efficacy of NK cell therapies, using a dual bioluminescent reporter gene system that is highly sensitive and quantitative. This *in vivo* approach is ideally suited for evaluating the complex biological processes of both cancer and NK cells in NK cell-based therapies (63). This molecular imaging technique could lay the basis for development of NK cell-based immunotherapies (46).

Teixeira et al. demonstrated that NK cells markedly reduced the tumor burden using a bioluminescent reporter-expressing xenograft/orthotropic tumor model. They showed that NK cells from healthy donors, upon activation with IL-2 and IL-15, indiscriminately kills both stem-like and differentiated bladder tumor cells (98). In addition to cell killing, NK cells shifted cancer stem-like cells toward a more differentiated phenotype, rendering them more susceptible to cisplatin, highlighting the benefits of a possible combined therapy. Zhu et al. demonstrated the therapeutic effects of a human NK cell line (NK92MI) for anaplastic thyroid cancer (CAL62) by bioluminescent imaging. They also developed a mouse model of lung metastasis with CAL62 cells expressing luciferase. NK92MI cells were injected through an intravenous route; this imaging approach showed the therapeutic effects of NK92MI within 2 days after the NK92MI injection (66).

May et al. also used bioluminescent imaging to demonstrate that SLP-76 (SH2 domain-containing leukocyte protein) knock-out (KO) NK cells cannot prevent tumor engraftment. They injected luciferase expressing CHO tumor cells, followed by injection of SLP-76 KO NK cells, Further imaging of tumor cells by bioluminescent imaging revealed that SLP-76 KO NK cells were incapable of killing tumor cells (59). Chandrasekaran et al. demonstrated an enhanced therapeutic effect of TRAIL-enriched NK cells for treatment of lymph node metastases of colon cancer by using bioluminescent imaging (SW620-expressing luciferase) (99).

Rygh et al. used dynamic contrast-enhanced MRI to detect therapeutic responses to NK cell immunotherapy in a rat model of glioblastoma and verified the usefulness of MRI for detecting early therapeutic responses to NK cell therapy combined with a mAb targeting Neuron-glia2 (NG2) to the tumor (69). Poli et al. visualized an increased therapeutic response by intra-tumoral treatment with NK cell therapy combined with a mAb targeting Neuron-glia2 (NG2) to U87MG glioblastoma by MRI, at 3 and 4 weeks *in vivo*, since the combined therapy led to massive recruitment of inflammatory cells into the tumor (100). In NK cell-based therapies, molecular imaging modalities can facilitate an in-depth exploration of NK cell behavior and tumor biology in specific microenvironments. Therefore, molecular imaging can play crucial roles in the development of NK cell-based immunotherapies against cancers.

## Advantages and Disadvantages for Imaging of NK Cells

Molecular imaging of cell-based cancer therapies has been an active area of investigation in both preclinical and clinical trials. With multi-modality imaging and probes, molecular imaging has spurred the development of new cell-based therapeutic strategies. *In vivo* monitoring of cell-based immunotherapies offers several advantages over traditional *ex vivo* methods that require animal sacrifice and histological analysis. Molecular imaging, for example, is non-invasive and allows for quantitative assessments of the biodistribution and effects of cell-based therapies over time. Optical imaging is a sensitive and suitable molecular imaging technology with multiple advantages for preclinical small animal experiments (63, 64) and is of particular value in mapping specific molecular mechanisms and non-invasively tracking specific cell types with optical reporters in living mice. These techniques are cheap, fast, and do not require radionuclides. Bioluminescent imaging using luciferase expression can reliably visualize as few as 100 cells in living animals (63). Recent progress in tomographic fluorescence fusion systems (fluorescence molecular tomography combined with anatomical imaging, such as FMT-CT and FMT-MRI) provide additional anatomical information to fluorescence-based imaging, which may overcome a critical limitation of optical imaging. However, optical imaging suffers from a low level of spatial resolution and tissue penetration, and it is still not suitable for clinical studies (64).

The immunogenicity of reporter proteins for optical imaging is an issue to consider. Fluorescence (GFP, RFP, and mCherry) and luciferase (*Renilla* luciferase and firefly luciferase)-associated gene products are foreign proteins that have the potential to be recognized by the immune system, which may lead to damage of the labeled immune cells. Nuclear medicine reporter genes existing in normal human cells, such as a sodium iodide symporter in thyroid follicular cells, are not immunogenic and can be used safely in the clinic (101, 102). Fluorescence microscopic imaging have a few drawbacks, which include photobleaching, the diffraction barrier of light, and the longer data acquisition times (79, 80).

A previous study had suggested the use of antibody-coated QDs for the tracking of NK cell-based cancer therapy, without compromising NK cell viability (70). However, delivery of imaging probes (ferumoxides and ferucarbotran) into cells by electroporation or microinjection damages the cell membrane and decreases cell viability (68, 90). This is in contrast to the ultimate goal of developing a method for cell tracking that is clinically deployable. MRI can provide excellent anatomical information in the form of three-dimensional tomographic images without a radiation hazard and is useful for tracking NK cell-based therapies in preclinical and clinical trials. However, MRI also can be applied to visualized cells after labeling with MR contrasts in *in vivo* models; however, its sensitivity is low. NK-92MI cells incubated with high concentration of SPIO nanoparticles showed decreased viability, and damaged or non-viable lysed cells release SPIO. This precludes accurate quantitative molecular imaging (68). Other disadvantages of direct labeling of NK cells with MR agents are the dilution-random distribution effect caused by cell division and signal persistence after cell death (103, 104). SPECT and PET imaging are very sensitive and have relatively good spatial resolution. SPECT and PET technologies allow quantitation and provide the potential to optimize cell-based therapies (92). Advantages and disadvantages of imaging modalities used in NK cells imaging are summarized in Table 2.

**Table 2:** Advantages and disadvantages of *in vitro* and *in vivo* imaging modalities used for natural killer (NK) cells.

Imaging modality		Advantages	Disadvantages	Applications			Reference
				*In vitro*	*In vivo*		
					Small animals	Human	
Optical imaging	FLI	Fast acquisition Easy dye labeling Inexpensive No radiation hazard	Low spatial resolution Poor tissue penetration Immunogenicity	Yes	Yes	No	(56, 61, 64, 95, 96)

	MI	Visualization of the NK immune synapse Visualizing the microarchitecture of NK cells	Immunogenicity Photobleaching Diffraction barrier of light Long data acquisition time Unsuitable for *in vivo* imaging	Yes	Yes	No	(48, 51, 71, 78, 79, 95–97, 100)

	BLI	Sensitive (100 cells) Suitable for preclinical small animal Inexpensive Fast No radiation hazard	Low spatial resolution Poor tissue penetration Immunogenicity	Yes	Yes	No	(63, 64)

Nuclear imaging	PET	Very sensitive Good spatial resolution Accurate quantitation	Slow acquisition Complicated labeling procedure Expensive	No	Yes	Yes	(92)

	SPECT	Very sensitive Good spatial resolution Accurate quantitation Possible long term *in vivo* monitoring (^111^In-oxine, 3 days)	Slow acquisition Complicated labeling procedure Expensive	No	Yes	Yes	(56, 61, 92)

MR imaging	MRI	Excellent anatomical information No radiation hazard	Low sensitivity Slow acquisition Ferumoxides labeling damages the cell membrane and decreases cell viability Release of MR contrasts from cells Expensive	No	Yes	Yes	(68, 83, 84, 98, 99)

*FLI, fluorescence imaging; MI, microscopic imaging; BLI, bioluminescence imaging; PET, positron-emission tomography; SPECT, single photon-emission computerized tomography; MRI, magnetic resonance imaging; SPIO, superparamagnetic iron oxide*.

## Perspectives

In the past two decades, numerous researchers and clinicians have recognized the significance of molecular imaging in gaining a fundamental understanding of NK cell biology at the cellular and molecular level in the treatment of cancer patients. Although we have gained considerable understanding of NK cell biology; many more questions about their biology remain unanswered. NK cell-based therapies for cancers are promising, but labeling and *in vivo* tracking of NK cells have predominantly been employed at the preclinical stage. Combining two or three of the most sensitive molecular imagining modalities may break through the current sensitivity limitation of *in vivo* NK cell imaging and may provide ways to track NK cells in living organisms and to monitor NK cells for a prolonged period of time. We believe that more unproven NK cell molecular mechanisms can be investigated using more sensitive and powerful molecular imaging modalities, and that major new discoveries in NK cell biology can be achieved through imaging observations in future.

In recent years, because of the advances in our understanding of gene regulation, tracer chemistry, and imaging technologies, many exciting possibilities are becoming practical in the imaging of immune cell kinetics and dynamics. Evolving imaging technologies will allow quantitative tracking of NK cells and will provide more refined information of vital biological processes through real-time cell tracking and imaging of cell–cell interactions in humans. Molecular imaging will not only help us to increase our knowledge but should also considerably speed the rate of discovery in the field of biological sciences.

## Conclusion

Molecular imaging techniques are able to provide new and better means for non-invasive, repeated, and quantitative *in vivo* tracking of NK cells. Although much information is readily available about the survival, biodistribution, and cytotoxicity to tumors in NK cell-based cancer treatment, significant gaps in our knowledge remain. Molecular imaging will continue to play a major role in answering key questions about clinical applications and will facilitate an understanding of the mechanisms underlying NK cell biology. Innovative advancements in molecular imaging technologies will contribute to improving NK cell-based cancer treatments by creating an understanding of and optimizing the use of NK cells in both preclinical and clinical settings.

## Author Contributions

PG and B-CA contributed to the conception, writing, and discussion of this review manuscript. PG wrote the initial draft of the manuscript. The final version of the manuscript was approved by both authors.

## Conflict of Interest Statement

The authors declare that the research was conducted in the absence of any commercial or financial relationships that could be construed as a potential conflict of interest.
